# The Spectrum of Endoscopic Ultrasound Intervention in Biliary Diseases: A Single Center's Experience in 31 Cases

**DOI:** 10.1155/2012/680753

**Published:** 2012-05-10

**Authors:** Siriboon Attasaranya, Nisa Netinasunton, Theeratus Jongboonyanuparp, Jaksin Sottisuporn, Teepawit Witeerungrot, Teerha Pirathvisuth, Bancha Ovartlarnporn

**Affiliations:** ^1^Department of Internal Medicine, Faculty of Medicine, Prince of Songkla University, Hat Yai, Songkhla 90110, Thailand; ^2^NKC institute of Gastroenterology and Hepatology, Faculty of Medicine, Prince of Songkla University, Hat Yai, Songkhla 90110, Thailand

## Abstract

*Background and Aim.* EUS-guided intervention (EGI) for biliary therapy has been increasingly used in recent years. This report aims to describe the spectrum and experience of EUS-guided interventions in biliary diseases in a single-tertiary center. *Methods.* All patients with EGI were analyzed retrospectively by retrieving data from a prospectively stored endoscopic database between January 2006 and September 2010. *Results.* There were 31 cases with EGIs (17 female, 14 male) with a mean age ± SD of 58.03 ± 16.89 years. The majority of cases (17/31; 55%) were ampullary or pancreatic cancers with obstructive jaundice. The major indications for EGI were obstructive jaundice (*n* = 16) and cholangitis (*n* = 9). The EGIs were technically successful in 24 of the 31 cases (77%). The success rate for the first 3 years was 8 of 13 procedures (61.5%) as compared to that of the last 2 years (16/18 procedures (89%); *P* = 0.072). Twenty-three of the 24 cases (96%) with technical success for stent placement also had clinical success in terms of symptom improvement. The complications were major in 4 (13%) and minor in 7 (23%) patients. *Conclusion.* The EUS-guided drainage for biliary obstruction, acute cholecystitis, bile leak, and biloma was an attractive alternative and should be handled in expert centers.

## 1. Introduction

Endoscopic ultrasound (EUS) guided cholangiography was firstly reported in 1996 [1]. EUS-guided interventions (EGIs) have been increasingly used in recent years [2, 3]. Many reports pertaining to EUS-guided biliary drainage in patients with failed ERCP, acute cholecystitis, and bilomas are available in the literature [4–36]. This report describes the spectrum and experience of EUS-guided interventions in biliary diseases in our center.

## 2. Methods

 This is a retrospective analysis of a prospectively stored endoscopic database from 2006 till September 2010. The study was approved by our institutional ethics committee. All of the endoscopic reports of EUS-guided interventions in biliary diseases were retrieved, and all of the endoscopic reports and medical records were reviewed. Data regarding the demographic profiles, indications, procedure types, technical success, clinical success, complications, and outcome of patients were analyzed.

 The main indications for EGIs of biliary diseases included patients with failed ERCPs for biliary therapy, patients with surgically altered anatomy preventing accessible ERCP, the unfit-for-surgery patients with cholecystitis not responding to medical treatment, and one patient with biloma requiring drainage. The EUS-guided intervention was performed using the Olympus EUS scope (GF-UCT160OL5, Olympus Medical, Tokyo, Japan) with a 3.7 mm working channel by two authors (S. Attasaranya and B. Ovartlarnporn). All of the procedures were carried out under conscious sedation with midazolam and pethidine supplemented with propofol where necessary, except one case (an 11-year-old girl) under general anesthesia. The common bile duct (CBD) was preferred as an access route in patients with distal bile duct obstruction, while the left intrahepatic duct was selected as an access point in patients with hilar obstruction, surgically altered anatomy, or narrowing of the pylorus preventing the EUS scope from passing into the proper position in the duodenum. The bile duct was punctured using a 19-gauge needle (Echotip Wilson-Cook Medical, Winston-Salem, NC). After confirmation of the proper position of the needle in the bile duct by aspiration for bile and contrast injection, a 0.035′′-guidewire (Jagwire Boston Scientific, Miami, FL) was inserted through the needle until several loops of the wire were coiled in the bile duct. The needle was removed, and the needle tract was dilated using an ERCP catheter followed by 6 Fr and 7 Fr dilating catheters (Soehendra dilation catheter, Wilson-Cook Medical) over the guidewire. A 7 Fr double-pigtail plastic stent (Zimmon biliary stent, Wilson-Cook Medical) or a partially covered metallic stent (Wallstent, Boston Scientific) was then inserted. If the needle tract dilatation using an ERCP catheter was unsuccessful, a needle knife (Microknife, Boston Scientific, Spencer, IN) using the endocut mode (VIO 300D, ERBE Elektromedizin GmbH, Tübingen, Germany) over the wire, was then introduced into the bile duct, and the tract was further dilated with 6 Fr and 7 Fr dilating catheters or an 8 mm biliary dilation balloon (Hurricane balloon catheter, Boston Scientific, Cork, Ireland). The procedures were performed in an out-patient setting in 4 cases and in an in-patient setting in 27 cases.

 The technical success was defined as a successful procedure with a properly placed stent, whereas the clinical success as the improvement of the symptoms intended to be treated by the procedure. The reduction of serum bilirubin level by a value greater than 50% measured after 2 weeks after the procedure as compared to the baseline value was defined as successful drainage for patients with obstructive jaundice.

The complications that required surgical interventions were defined as major complications, and those that recovered spontaneously or responded to medical therapy or minimally invasive procedures were defined as minor complications.

## 3. Results

 A total of 31 cases (17 female and 14 male) underwent EUS-guided interventions during the period under investigation. The mean age ±  SD was 58.0 ± 16.9 years, with a range of 11 to 88 years. The median follow-up time in the 28 patients with available follow-up data was 3.4 months, with a range of 0.3–21.5 months. Fourteen patients died, ten patients were still alive, and seven patients defaulted during the followup.

The diagnoses of the patients comprised periampullary or pancreatic cancer in 17, gastric cancer in 1, duodenal cancer in 1, pancreatic inflammatory pseudotumor in 1, metastatic cancer in 2, choledochojejunostomy stenosis in 3, gallstone with cholecystitis in 1, post ERCP cholecystitis in 1, CBD stone in 1, bile leak in 1, hilar cholangiocarcinoma in 1, and biloma with postlaparoscopic cholecystectomy bile leak in 1.

The indications for endoscopic drainage were obstructive jaundice in 16, cholangitis in 9, cholecystitis in 2, choledochojejunostomy stenosis in 1, replacement of percutaneous transhepatic biliary drainage (PTBD) in 1, bile leak in 1, and biloma in 1 (Table 1). The reasons for EUS-guided drainage were failed ERCP for biliary cannulation in 14, inaccessible ERCP due to luminal stenosis secondary to tumor invasion of gastric antrum or duodenum in 10, surgically altered anatomy in 4, acute cholecystitis with unfit condition for surgery in 2, and biloma in 1.

The EUS-guided interventions (EGIs) were technically successful in 24 of 31 (77%) cases. Twenty-three (96%) of those with technical success for EGIs had clinical success in terms of symptoms improvement also. There were 16 hepaticogastrostomies (HG) with 3 failures, 9 choledochoduodenostomies (CD) with 3 failures, 4 cholecystoduodenostomies (CHD) with 1 failure, 1 antegrade placement of metallic stent in CBD through the duodenal wall, and 1 biloma drainage performed. Covered metallic stents were inserted in 4 patients and plastic stents in 22 others (Table 2). The failure rates in the HG and non-HG procedures were 3 in 16 (19%) and 4 in 15 (27%), respectively, but the difference was not statistically significant. There were 5 failures out of 13 procedures done in the first 3 years (38%) and 2 failures out of 18 procedures in the last 2 years (11%); the difference was not significant (*P* = 0.072). The mean hospital stay in the 27 patients on whom procedures were performed in an in-patient basis was 16.8 ± 28.6 days. In the subgroup analysis to compare the mean hospital stay between the patients with successful (*N* = 20) and those with failed procedures (*N* = 7), no statistical difference between the two groups was observed (17.3 ± 33.3 versus 15.1 ± 6.3 days; *P* = 0.78).

 There were 11 complications in the 31 procedures (35%) as shown in Table 3. The complications were major in 4 (13%) and minor in 7 (23%) cases. In subgroup analysis, 2 major and 2 minor complications occurred in the patients with EUS-guided CD, while 1 major and 5 minor complications occurred in those with EUS-guided HG. There was no statistical difference of complication rate between the two groups (*P* = 0.527). The four major complications included delayed migration of the stent out of the bile duct leading to bile peritonitis requiring surgery for biliary diversion in 1, malposition of stent deployment resulting one tip of the stent located in intraperitoneum requiring surgery to retrieve the stent into a proper position in 1, perforation induced by the EUS scope requiring surgical repair in 1, and bile peritonitis after failed EUS-guided CHD requiring surgical intervention in 1. All but one patient recovered following surgical intervention. The patient with bile peritonitis after the failed EUS-guided CHD had a stormy, deteriorating postoperative course. The patient was eventually referred to a local facility for best supportive care in accordance with the patient and his family's wish. The minor complications included severe abdominal pain with pneumoperitoneum that subsided with conservative treatment in 1, postprocedure fever in 3, minor abdominal pain in 1, infected retrogastric collection resolved by medical therapy with percutaneous drainage in 1, and self-limited bleeding at the puncture site in 1. The complication rate for the first 3 years was 7 of 13 procedures (54%), which trended to be higher than that for the last two years (4 of 18 procedures; 22.2%) but with no statistical insignificance (*P* = 0.087).

Stent exchange was required in 3 patients during the followup. One patient with metastatic gastric cancer had the stent occluded at 3-month intervals and required another EUS-guided metallic stent placement. Another patient with recurrent pancreatic cancer after Whipple's operation underwent a biliary plastic stent exchange 2 months after the procedure. The third patient with a stricture during hepaticojejunostomy was treated with percutaneous placement of a metallic stent elsewhere, but the stent was blocked, leading to cholangitis. The stent was partially removed using single-balloon enteroscopy followed with EUS-guided HG with a metallic stent placement. Cholangitis resolved, but the new stent was occluded 3 weeks later. The repeated endoscopy revealed that the tip (gastric side) of the stent was found to be too long and submersed under gastric juice. Trimming of the stent tip with argon plasma was performed, and the stent was then cleaned by repeated balloon sweeping. However, multiple episodes of stent occlusion still recurred, requiring additional plastic stent insertion. The patient defaulted after a 9-month followup following the first procedure. Three patients, two with acute cholecystitis and one with biloma, had their stents removed after the resolution of symptoms. All of the stents in the remaining 17 patients with successful procedures were still functioning at the time of the last followup.

## 4. Discussion

 The EUS-guided interventions for biliary diseases were demonstrated to be an attractive alternative in our report. The overall technical success rate in our study was in the boundary of the 50–100% range that is reported in the literature [4–36]. In the study by Itoi et al. [16], the technical success rate for EUS-guided HG ranged from 91–100%, and the success rate of EUS-guided CD ranged from 50–100%. The 81% success rate for the former and the 67% success rate for the latter in our series were similar to the data reported in the literature. The higher success rate for EUS-guided HG suggested that it may be easier to perform than EUS-guided CD, which was in agreement with other reports [34]. The failure rate of 38% in the initial 3 years compared with that of 11% in the last 2 years suggested that a learning curve period was required to improve technical skill. Notably, our success rate of 89% in the last 2 years was comparable to the ranges reported in other series [16].

 The EUS-guided drainage of the gallbladder (GB) in our report showed the potential use of this approach in treating selected patients with acute cholecystitis. Only a few reports with a small number of patients have reported the utility of EUS-guide drainage of the GB in cholecystitis [21–24, 32, 33]. In the literature review by Itoi et al. [22], 24 cases were reported with EUS-guided drainage of the GB in acute cholecystitis, with clinical success in all cases and a 25% complication rate. Recently, Itoi et al. have reported a case series of 5 patients with acute cholecystitis successfully treated via EUS-guided GB drainage using novel fully covered metallic stents designed to have bilateral anchor flanges [37]. Interestingly, the stent stayed fully patent for 12 months in a patient whose GB stent remained dwelling. The benefit of long-term GB stenting in this setting, however, needs to be determined by further study.

 The EUS-guided drainage of the GB without acute cholecystitis was performed on 2 patients in our report. One patient with failed ERCP intervention causing subsequent bile leak showed resolution of the leakage following EUS-guided GB drainage. The other patient with obstructive jaundice from malignant distal bile duct stricture, whose HG and CD could not be performed due to the small degree of upstream duct dilation, failed the EUS-guided drainage of the GB and developed bile leak from the punctured GB leading to a dismal outcome. In detail, the failure in this patient occurred due to the dislodgement of the proximal tip of the stent out of the GB during stent deployment. Subsequent attempts at EUS-guided puncturing of the GB for another stent placement were unsuccessful presumably due to the collapsed GB following the leak. To the best of our knowledge, there has been no report regarding EUS-guided drainage of the GB without acute cholecystitis. For noninflamed GB, EUS-guided drainage should be done with caution since the GB is mobile; thus, the procedure may be more challenging, and the risk of bile leakage into the peritoneum is theoretically higher than that involving cases of inflamed GB, in which the surrounding adhesion may prevent bile leakage into the peritoneal cavity. Further refinement regarding the techniques and development of accessories for the procedure are needed before EUS-guided drainage of the GB can be accepted as a standard option.

 EUS-guided drainage of the bile duct in patients with surgically altered anatomy was carried out in 4 patients and all of them experienced technical success. One of our patients had an ERCP performed using a single balloon enterosope, but only partial removal of the blocked metallic stent was accomplished. The EUS-guided drainage provided some additional treatment, but it did not completely solve the problem of recurrent stent occlusion. Two of our patients underwent EUS-guided bile duct drainage for anastomosis stricture. In one of the two patients, the stricture was dilated with a biliary balloon, and a concomitant bile duct stone was successfully pushed through the anastomosis into the jejunal limb using a balloon resulting in improved symptoms. Another patient underwent an HG and waited for the tract to mature before tract dilatation to deal with the stricture. In another patient, the EUS-guided drainage was used to replace the preexisting PTBD to improve quality of life, but the stent became malfunctional at 4 weeks after insertion. A PTBD was eventually required for biliary drainage. The ultimate role of EUS-guided drainage in patients with surgically altered anatomy as compared with ERCP by a balloon enteroscope or a percutaneous approach needs further study for clarification.

 One patient with biloma was successfully drained by the EUS-guided approach in this report. Shami et al. has reported on EUS-guided drainage of biloma with a clinical response in 5 patients [31]. EUS-guided drainage of bilomas is technically feasible, appears safe, and provides an attractive alternative to other treatment modalities.

 The overall complication rate of EUS-guided intervention of 35% in this report was within the range reported in the literatures [4–36]. However, the complication rate of 0–36.3% reported in the literatures may be an under estimation, since series with unfavorable outcome are more likely to be unreported. Most of the complications in our series were minor, but the 4 (13%) major complications are of concern. Three out of 4 major complications occurred following technical failure (Table 3). Notably, two of the major complications were related to stent displacement; one was related to delayed dislodgment of a double-pigtail plastic stent out of the common bile duct in the patient undergoing an EUS-guided CD, and the other was related to the improperly placed metallic stent with the proximal end dislodged into the intra-abdominal cavity. In order to prevent stent dislocation/migration, some experts have suggested that a relatively longer plastic stent is preferable, particularly when placing via a HG approach. The shortening ratio of the metallic stent should also be taken into account when placing the braided-type metallic stent [38].

 The complication rate declining from 53% in the first 3 years to 22% in the last 2 years might imply that more experience resulted in a more favorable outcome.

The longer-than expected hospital stay (mean 16.8 ± 28.6 days) in our study was due to multiple contributing factors mostly unrelated to the procedures per se. Most patients required concomitant therapy for their underlying medical conditions as well as chemotherapy for the underlying malignancies.

In conclusion, our data suggest that EUS-guided biliary intervention is feasible and an attractive alternative tool providing biliary drainage in selected patients and should be performed by experienced endoscopists.

## Figures and Tables

**Table 1:** Indications for EUS-guided interventions.

Indication	Number of cases
Obstructive jaundice	16
Cholangitis	9
Cholecystitis	2
Choledochojejunostomy stenosis	1
PTBD replacement^f^	1
Bile leak	1
Biloma	1

^f^Percutaneous transhepatic biliary drainage.

**Table 2:** Type of drainage, success rate, and type of stent inserted.

Type of procedure	No.	Success
Hepaticogastrostomy	16	13
Choledochoduodenostomy	9	5
Cholecystoduodenostomy	4	3
Biloma drainage	1	1
Antegrade placement of metallic stent in CBD through the duodenal wall	1	1

Total	31	24

Type of stent		

Plastic, double pigtail	22	
Metallic, partially covered	4	

**Table 3:** Complications, treatment, and outcomes.

	Number	Outcome
Major complications in technical success		
Stent slipped off 2 days later	1	Surgery done, recovered
Major complications in technical failure		
Metallic stent deployed outside gastric wall	1	Surgery done, recovered
Duodenal perforation	1	Surgery done, recovered
Bile peritonitis	1	Surgery done, deteriorated
Total major complications	4	
Minor complications in technical success		
Severe abdominal pain	1	Resolved with medical treatment
Postprocedure fever	2	Recovered with antibiotics
Mild abdominal pain	1	Recovered
Retrogastric collection	1	Recovered with PCD^+^
Minor complications in technical failure		
Bleeding	1	Stopped spontaneously
Postprocedure fever	1	Recovered with antibiotics
Total minor complications	7	
Total overall complications	11	10 with recovery

^+^Percutaneous drainage.
